# Detection of musculoskeletal inflammatory lesions in patients with chronic chikungunya infection using 3T whole-body magnetic resonance imaging

**DOI:** 10.1590/0037-8682-0090-2024

**Published:** 2024-05-27

**Authors:** Aline Serfaty, Silvana Mendonça, Clarissa Canella, Edson Marchiori

**Affiliations:** 1 Universidade Federal do Rio de Janeiro, Rio de Janeiro, RJ, Brasil.; 2 Medscanlagos Diagnóstico por imagem, Cabo Frio, RJ, Brasil.; 3 Clínica de Diagnóstico por Imagem CDPI, Rio de Janeiro, RJ, Brasil.; 4 Universidade Federal Fluminense, Niterói, RJ, Brasil.

**Keywords:** Musculoskeletal diseases, Chikungunya infection, Magnetic resonance imaging, Polyarthralgia, Tenosynovitis

## Abstract

**Background:**

Musculoskeletal inflammatory lesions in chronic Chikungunya virus (CHIKV) infection have not been thoroughly assessed using whole-body magnetic resonance imaging (WBMRI). This study aimed to determine the prevalence of these lesions in such patients.

**Methods:**

From September 2018 to February 2019, patients with positive Chikungunya-specific serology (Immunoglobulin M/Immunoglobulin G anti-CHIKV), with a history of polyarthralgia for > 6 months prior to MRI with no pre-existing rheumatic disorders, underwent 3T WBMRI and localized MRI. The evaluation focused on musculoskeletal inflammatory lesions correlated with chronic CHIKV infection. Pain levels were assessed using a visual analogue scale on the same day as WBMRI.

**Results:**

The study included 86 patients of whom 26 met the inclusion criteria. All patients reported pain and most (92.3%) categorized it as moderate or severe. The most common finding across joints was effusion, particularly in the tibiotalar joint (57.7%) and bursitis, with the retrocalcaneal bursa most affected (48.0%). Tenosynovitis was prevalent in the flexor compartment of the hands (44.2%), while Kager fat pad and soleus edema were also observed. Bone marrow edema-like signals were frequently seen in the sacroiliac joints (19.2%). Most WBMRI findings were classified as mild.

**Conclusions:**

This study represents the first utilization of 3T WBMRI to assess musculoskeletal inflammatory disorders in chronic CHIKV infection. The aim was to identify the most affected joints and prevalent lesions, providing valuable insights for future research and clinical management of this condition regarding understanding disease pathophysiology, developing targeted treatment strategies, and using advanced imaging techniques in the assessment of musculoskeletal manifestations.

## INTRODUCTION

Chikungunya virus (CHIKV) infection is an arboviral disease caused by the alphavirus CHIKV and transmitted by *Aedes aegypti* and *A. albopictus* mosquitoes. It was first described in Tanzania in the 1950s, with a small number of outbreaks being subsequently reported in Asia and Africa. In 2005, a severe outbreak reached La Réunion island in the Indian Ocean, with 225,000 cases reported. Since then, several outbreaks have been reported, especially in Central and South America^1^ ^-^ ^3^.

In Brazil, CHIKV was first identified in 2014 with a yearly growing incidence and with a peak incidence of 60 new cases/100,000 inhabitants recorded in 2018 in the state of Rio de Janeiro. Laboratory tests, including molecular or serological tests, can confirm the diagnosis and differentiate CHIKV infection from other endemic diseases transmitted by the same vector, including Dengue and Zika^3^ ^,^ ^4^.

Three stages may be observed over the course of the disease: 1) an acute stage (from 1 to 2 weeks) that may manifest with sudden onset of high fever and symmetric polyarticular joint pain (more frequently in the hands, wrists, ankles and feet), associated with headache, myalgia and a maculopapular rash over the trunk and extremities; 2) a subacute stage (up to 3 months) where joint symptoms predominate; 3) and the chronic stage (> 3 months, may last years after acute infection) when symmetric distal polyarthralgia persists or relapses^1^ ^,^ ^2^ ^,^ ^5^. 

A small number of studies have demonstrated musculoskeletal imaging findings in chronic CHIKV infection, most of them based on ultrasound findings^6^ ^-^ ^8^. The purpose of our study was to describe the prevalence of musculoskeletal inflammatory lesions with whole-body magnetic resonance imaging (WBMRI) in patients with chronic CHIKV infection.

## METHODS

The institutional review board of MedScanLagos approved this retrospective study and each patient formally consented to take part (Certificate of Submission for Ethical Appraisal: 21749613.0.000.5259). Written informed consent was obtained from all patients.

### ● Study Participants

We performed a retrospective study from September 2018 to February 2019 involving patients diagnosed with positive Chikungunya-specific serology (IgM/IgG anti-CHIKV) and with a history of chronic polyarthralgia who were followed in a rheumatology outpatient clinic in the state of Rio de Janeiro, Brazil. Our inclusion criteria consisted of positive Chikungunya-specific serology and a history of polyarthralgia for > six months before MRI. Exclusion criteria were previous rheumatic diseases, septic arthritis, claustrophobia, and age > 70 years since older individuals usually have a higher prevalence of comorbidities or musculoskeletal symptoms that may be related to age or other underlying conditions, leading to possible misinterpretations of imaging findings, and increasing the likelihood of confounding factors. Among 86 selected patients, 35 were not reachable or did not wish to participate.

### ● Clinical Assessment

We assessed demographic and clinical characteristics of the study population, including age; sex; time period, location and severity of symptoms. Pain duration ranged from 6 to 24 months. On the same day of MRI, patients were asked to evaluate their pain using a visual analogue scale comprised of a horizontal line, 10 centimeters in length, with two verbal descriptors in the extremities (mild and severe pain) and one in the middle of the scale (moderate pain)^9^ ^,^ ^10^. 

### ● MRI Parameters

**FIGURE 1:**
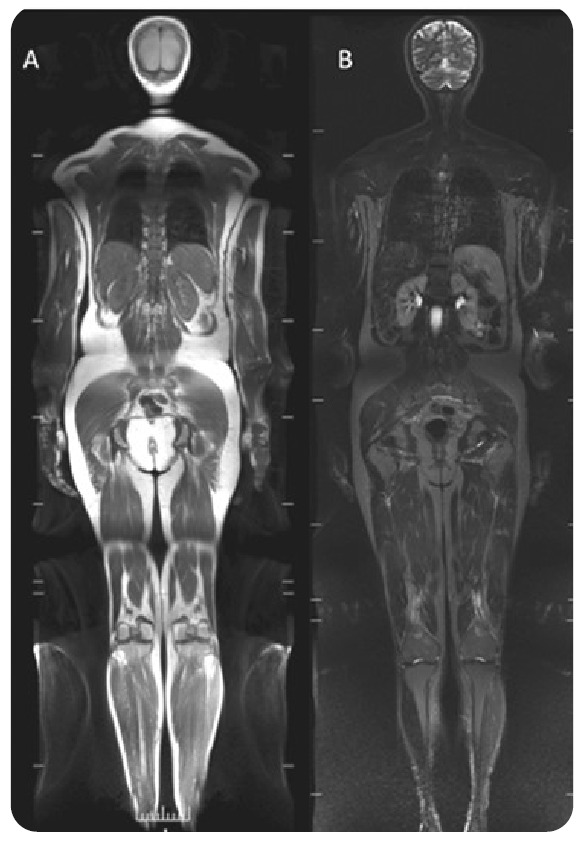
WBMRI protocol with T1 **(A)** and STIR **(B)** weighted images of the whole-body in coronal plane.

MRI was performed with a 3.0-T imaging unit (GE Medical Systems, Milwaukee, WI, USA) using the following coils: a 20-channel head/neck, an 18-channel body array, a 32-channel spine array and 16-channel flex array. The imaging protocol included T1- and STIR-eighted images of the whole-body in the coronal plane (Figure 1). Sagittal MRI of the whole spine, coronal images of the sacroiliac joints, and localized multiplanar images of wrists, hands, ankles and feet in STIR- and fat-suppressed T2 (FS T2) sequences were also obtained. 

### ● Image Processing and Analysis

Two musculoskeletal radiologists (A.S. and S.M., each with 11 years of experience) evaluated MRI examinations using GE Advantage Workstation Volume Share 7 (v.AW4.7) and the OsiriX software package (v.3.8.1; OsiriX Foundation, Geneva, Switzerland). Both radiologists were blinded to annotations, clinical findings, and evaluated all patients independently. Before imaging review, a training session was conducted with use of a group of WBMRI examinations (different to those of the study) to standardize the criteria for diagnosing inflammatory musculoskeletal findings.

WBMRI and localized images were used to identify inflammatory musculoskeletal disorders in the following peripheral joints: shoulders, wrists, hands, hips, knees, ankles and feet. We also searched for inflammatory lesions in axial joints by evaluating vertebral bodies and posterior parts of the cervical, dorsal, and lumbar segments of the spine and sacroiliac joints.

The following lesions were evaluated in each of the aforementioned joints and were subsequently classified as mild, moderate, or severe^11^ ^,^ ^12^:

- joint effusion, defined as an increased amount of fluid in the synovial compartment of a joint better depicted in STIR and/or fat-suppressed proton density (FS T2) images;
- tenosynovitis defined as peritendinous effusion seen on axial sequences in at least 3 consecutive slices in STIR and/or FS T2 images;
- bursitis defined as fluid collected within the bursa resulting in pathological enlargement of the bursa observed in STIR and/or FS T2 images;
- muscle edema defined as increased signal intensity within the muscles in STIR and/or FS T2 images
- Kager fat pad edema defined as increased signal intensity in those locations in STIR and/or FS T2 images;
- bone marrow edema-like signal intensity, defined as a hyperintense lesion in STIR and/or FS T2 and hypointense in T1 with ill-defined margins within the trabecular bone;

### ● Statistical Analysis

Demographic and clinical characteristics of the study population were described using summary statistics (mean, range, and standard deviation). Absolute and relative frequencies were used to describe pain categories on a pain scale.

Imaging findings were classified as mild, moderate, and severe. The prevalence of imaging findings in each of the joints was calculated for each evaluation by the main reader (radiologist #1) and for the evaluation of radiologist #2 using absolute and relative frequencies.

We assessed inter-reader agreement using Kappa coefficients (with respective 95% confidence limits). Intra-observer analysis was calculated for both reads of radiologist #1 (reads were a month apart) and inter-observer analysis was performed by paring the first read of radiologist #1 and the read of radiologist #2 with results showing concordance.

Chi-squared trend tests were used to evaluate the association between the severity of the reported pain and imaging findings in distal peripheral joints (hands, wrists, ankles and feet) where pain was more frequently reported.

We performed all statistical analyses using IBM-SPSS for Windows v.22.0, Armonk, NY (2013). The tests were performed with a 5% statistical significance level.

## RESULTS

Of the 86 initial patients, six were excluded because of previous rheumatic diseases; one was excluded because of septic arthritis involving the hip, three were excluded because of claustrophobia; and fifteen were excluded because age was > 70 years. Among the 86 selected participants, 35 were not reachable or did not wish to participate. Twenty-six participants met the inclusion criteria. Ages ranged from 29 to 67 years and the mean patient age (standard deviation) was 52.1 (9.8) years; 21 of the 26 (80.7%) patients were women. 

Pain was reported by all patients. Most of the patients presented with severe pain (53.8%). Most of the patients presented with symmetric polyarthralgia in hands and feet (13/26 patients; 50%), followed by the shoulders and ankles (8/26 each; 30.7%). In the axial joints, one patient reported pain in the cervical spine while three patients reported back pain.

No association was observed between severity of the reported pain and the presence of imaging findings in distal peripheral joints (hands, wrists, ankles and feet) where pain was more frequently reported (p-value = 0.125 for joint effusion; p-value = 0.400 for bursitis; p-value = 0.599 for tenosynovitis; p-value = 0.332 for bone marrow edema-like signals; p-value = 0.594 for muscle edema; and p-value = 0.868 for Kager fat pad edema).

There was almost perfect agreement between the two reads of radiologist #1. Agreement between the two reads of radiologist #1 was perfect for the ankles and sacroiliac joints (Kappa = 1), and almost perfect in other evaluated sites (shoulders (Kappa = 0.910; 95% CI: 0.788 to 1), wrists (Kappa = 0.867; 95% CI: 0.722 to 1), hands (Kappa = 0.883; 95% CI: 0.756 to 1), hips (Kappa = 0.936; 95% CI: 0.811 to 1), knees (Kappa = 0.961; 95% CI: 0.887 to 1), and feet (Kappa = 0.920; 95% CI: 0.812 to 1)). The agreement between the reads of radiologists #1 and #2 was perfect for the shoulders, wrists, hips, knees, and sacroiliac joints (Kappa = 1), almost perfect for the hands (Kappa = 0.802; 95% CI: 0.637 to 0.967), substantial in the feet (Kappa = 0.735; 95% CI: 0.559 to 0.911), and reasonable in the ankles (Kappa = 0.381; 95% CI: -0.152 to 0.914). This was because 92.3% of the ankles revealed some finding, and there were only 3 cases where the observers disagreed. The results of the main reader were considered for the analyses.

As most of the imaging findings were classified as mild for all of the assessments (with only a small number categorized as moderate or severe), for subsequent analyses they were recorded as present or absent.

### ● Frequency and distribution of musculoskeletal lesions 

**FIGURE 2:**
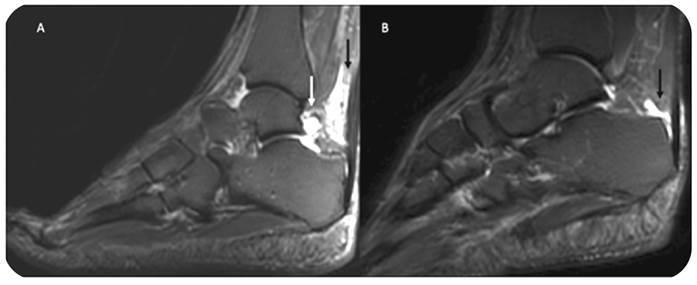
**A.** 61-year-old female with severe pain in the knee and ankles for 7 months with use of steroids and methotrexate. Sagittal STIR depicts joint effusion in the posterior subtalar joint (white arrow) and Kager fat pad edema (black arrow). **B.** 38-year-old female with pain in shoulders, hands, legs and feet for one year, with use of steroids and methotrexate. Sagittal STIR shows retrocalcaneal bursitis (black arrow).

The most common imaging finding in all joints was joint effusion, present in 25 of the 26 patients (96.1%) (Figure 2, Supplementary Table 1). This finding was observed more frequently in the ankles (n = 32/52; 61.5%), with the tibiotalar joint being the most affected site (n = 30/52 57.7%). Other frequent locations were the knees (n = 24/52; 46.2%), hands and feet (n = 20/52 each; 38.5%), and wrists (n = 15/52; 28.8%).

Bursitis was mostly observed in the retrocalcaneal bursa, recorded in 25 (48.1%) joints (Figure 2). It was also observed in the intermetatarsal bursas in 16 feet (30.8%), in the shoulders (n = 13/52; 25.0%) and in the hips (n = 8/52; 15.4%).

**FIGURE 3:**
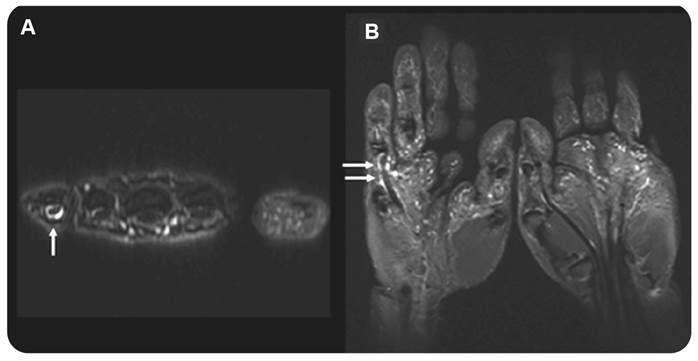
61-year-old female with pain in hands and feet for one year with use of nonsteroidal anti-inflammatory drugs. Axial T2 fat-suppressed weighted image of the right hand and coronal STIR of the hands show tenosynovitis of the flexor tendon of the fifth finger.

**FIGURE 4:**
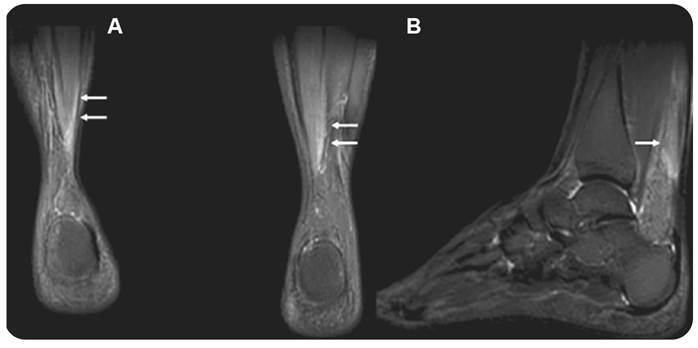
49-year-old female with pain in arms, hands, ankles and feet for a year, in treatment with steroids. Coronal **(A)** and sagittal **(B)** STIR of the ankles depicts edema in the distal portion of the soleus (arrows).

Tenosynovitis was present in 21 patients (80.7%). It was recorded in the flexor compartment of the hands in 23 joints (44.2%) (Figure 3) and in the lateral compartment of the ankle in 18 (34.6%). In addition to tenosynovitis in the ankle, Kager fat pad edema was observed in 34 joints (65.3%) of 20 patients (76.9%) (Figure 2) while muscle edema was only observed in the soleus of 8 ankles (15.4%) in 5 patients (19.2%) (Figure 4). 

Bone marrow edema-like signals were recorded in 11 of the 26 patients (42.3%). The sacroiliac joint was the most frequent location, with 10 of 52 joints involved (19.2%). Bone marrow edema-like signal in the vertebral corners was observed in 3 patients (11.5%) in the lumbar segment, while in the cervical and dorsal segments, it was recorded in 1 patient each (3.8%).

**FIGURE 5:**
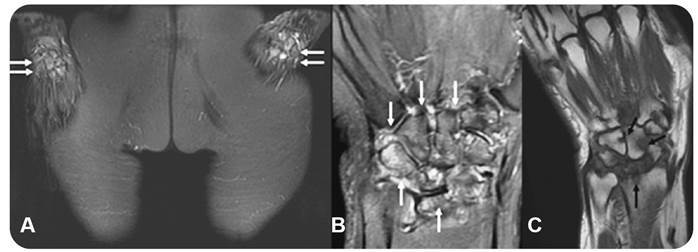
64-year-old female with severe pain in wrists, hands and ankles for two years. Current with no treatment. Coronal STIR **(A)**, coronal PD-weighted **(B)** and T1-weighted **(C)** images depict bilateral bone marrow edema of the carpal bones (white arrows) and erosions (black arrows).

Interestingly, one patient presented with bilateral bone marrow edema-like signals of the carpal bones associated with distal radioulnar effusion similar to that which is observed in rheumatoid arthritis (Figure 5). This patient also presented bone marrow edema-like signals involving the sacroiliac joints, in the calcaneal sulcus, and in the lateral process of the talus together with sinus tarsi synovitis.

## DISCUSSION

Musculoskeletal manifestations of CHIKV infection have affected a wide range of those infected, with symmetric polyarthralgia being the most commonly reported symptom, particularly involving the wrists, hands, ankles, and feet^2^ ^,^ ^3^ ^,^ ^13^ ^,^ ^14^ ^,^ ^15^. In our study, most patients reported pain in the extremities, especially in hands and feet, in concordance with previous reports in the literature. All patients reported pain at least in one joint on the same day of the MRI, with 92.3% (n = 24) rating it as moderate or intense on a visual pain rating scale.

Pain has an important role in the course of CHIKV infection as it can cause disabilities, which is more significant in economically active patients, resulting in long-term absence from work and loss of quality of life^14^ ^,^ ^16^ ^,^ ^17^. Several case reports have described pain as moderate or severe in patients with CHIKV infection. Although Brazilian guidelines for CHIKV infection^18^ have recommended the use of a visual analogue scale for the assessment of pain at all stages of the disease, we identified only one study^19^ in which a visual analogue scale was used to assess pain in the setting of subacute and chronic CHIKV infection, with most of the patients reporting pain as moderate or severe, similar to our study. Although we did not find a direct association between the severity of pain symptoms and imaging findings on WBMRI in CHIKV-infected individuals, we believe MRI should be performed. It allows for the identification of the most common musculoskeletal lesions involving this infection and is useful in differentiating it from other rheumatic diseases or infections, contributing to a more accurate diagnosis and appropriate treatment. Additionally, MRI can assess disease progression, helping to monitor these changes over time and adapt treatment strategies accordingly.

WBMRI has been used to detect axial and peripheral inflammatory and structural lesions in patients with rheumatic diseases such as axial spondyloarthritis and psoriasis^11^ ^,^ ^12^ ^,^ ^20^. However, according to our knowledge, this is the first study to evaluate musculoskeletal inflammatory lesions in patients with chronic CHIKV infection. 

Joint effusion was the most common musculoskeletal finding in our cohort, mainly observed in the ankles in concordance with what has been previously published in the literature^3^ ^,^ ^7^ ^,^ ^8^ ^,^ ^21^ ^-^ ^23^. Mogami at al^6^ found similar results involving the ankles of 52 patients with chronic CHIKV infection, with the tibiotalar joint being the most commonly affected site. Joint effusion was also the most common finding in a longitudinal study performed by Manimunda et al.^8^ with 20 patients who underwent MRI in the chronic stage of the disease. 

Bursitis was a very frequent imaging finding in our study, mostly observed in the retrocalcaneal region. Although Mogami et al.^6^ also recorded retrocalcaneal bursitis in their study, we found a much higher percentage of it. Tenosynovitis has been frequently reported in CHIKV infection, especially in the wrists, hands, ankles and feet, regardless of the stage of the disease^6^ ^,^ ^8^ ^,^ ^9^ ^,^ ^22^ ^,^ ^24^. In our study, it was mainly observed in the flexor compartment of the hands in concordance with another Brazilian study^22^ performed with patients in the subacute and chronic stages of the disease, and with a reported prevalence of 70%. We also recorded a high prevalence of tenosynovitis involving the ankles, mostly around the peroneus tendon and posterior tibial tendon, as has been demonstrated in other studies^6^ ^,^ ^25^. The ankle was also the only location where muscle edema was observed in our study. Little is understood concerning the pathophysiology of CHIKV infection; however, it has been suggested that the virus may persist in the skeletal muscles and myotendinous insertions perpetuating myositis, thereby leading to muscle edema^7^ ^,^ ^26^ ^,^ ^27^. This finding has been reported for the long flexor of the hallux and in the thenar and hypothenar musculature^6^ ^,^ ^9^. We recorded it only in the soleus muscle. 

We found a much a greater percentage of Kager fat pad edema comparing to that which has been previously reported^6^. One possible explanation is that we used MRI to assess the whole body while most other studies used ultrasonography which is less accurate and operator dependent. Although Kager fat pad edema may be observed without soleus edema, the contrary was not recorded in our findings.

Bone marrow edema-like signals have been reported in chronic stages of CHIKV infection, mainly in the sacroiliac joint, as observed in our study^21^. Although Manimunda et al.^8^ did not specify the sites of edema-like signal intensity, they described this finding in 35% of their cases, a percentage slightly inferior to that recorded in our study.

Rheumatoid arthritis-like signals was observed in one patient in our study. Persistent polyarthralgia and arthritis have been reported in patients with CHIKV infection, with a variable prevalence of those who meet the American College of Rheumatology criteria for rheumatoid arthritis. Erosive lesions were also more commonly seen in this type of presentation^3^ ^,^ ^5^ ^,^ ^6^ ^,^ ^24^ ^,^ ^28^ ^-^ ^31^.

A recent study has been conducted to develop and validate a WBMRI score for inflammation in peripheral joints and entheses in inflammatory arthritis (MRI-WIPE) using a semiquantitative scale of 0-3 (none/mild/moderate/severe)^11^. The imaging findings in our study were similarly stratified and although most of the patients reported significant pain, the vast majority of MRI findings were rated as mild, revealing a discrepancy between clinical and imaging findings in the chronic stage of CHIKV infection. We also did not find any statistical association between the degree of pain and imaging findings involving the most painful joints (wrists, hands, ankles and feet) (p-value > 0.05).

There were certain limitations to our study. Firstly, laboratory findings [C-reactive protein (CRP) levels and erythrocyte sedimentation rates (ESR)] were not available for all patients on the same day of WBMRI. CRP and ESR are commonly used as markers of inflammation, as they are typically elevated in response to inflammatory processes, including infections. In the context of chronic CHIKV infection, the presence or absence of elevated CRP and ESR levels can provide valuable insights into ongoing inflammatory responses and help monitor the effectiveness of treatment. The inclusion of CRP and ESR measurements in the inclusion criteria could have enhanced our understanding of the inflammatory processes involved in CHIKV infection. Secondly, contrast media were not employed to evaluate synovitis. Post-contrast images can improve the visualization of synovial inflammation and enhance the detection of subtle changes in the synovium. This allows for a more accurate assessment of disease activity, as it can differentiate between active inflammation and chronic changes involving the synovium. Additionally, it can also help to monitor treatment responses by assessing changes in synovial inflammation over time.

In conclusion, according to our knowledge, this study represents a pioneering effort in utilizing 3T WBMRI to evaluate musculoskeletal inflammatory disorders in CHIKV-infected patients. By identifying prevalent lesions and frequently affected joints, we have provided valuable insights into the disease's musculoskeletal manifestations. Future studies should focus on defining WBMRI's role in delineating distribution patterns of this disease, which could further enhance our understanding and management of chronic CHIKV infection. Moreover, the comprehensive assessment provided by WBMRI highlights its potential for improving disease classification, distinguishing CHIKV infection from other rheumatic conditions, and guiding tailored treatment strategies. The correlation of WBMRI findings with clinical symptoms could lead to the development of novel pain or complication scoring systems, facilitating better disease monitoring and management.
