# Supplementary material for: Detection of musculoskeletal inflammatory lesions in patients with chronic chikungunya infection using 3T whole-body magnetic resonance imaging
Source: Rev Soc Bras Med Trop. 2024 May 27;57:e00406-2024. doi: 10.1590/0037-8682-0090-2024 (PMC11136507; doi:10.1590/0037-8682-0090-2024)
Supplement: Supplementary file 1 [file 1678-9849-rsbmt-57-e00406-2024-supp1.pdf]

SUPPLEMENTARY TABLE 1: Frequency of Lesions in the Peripheral and Axial Joints on MRI.

Variable	Central and Peripheral Joints										
	Shoulders (n = 52)	Wrists (n = 52)	Hands (n = 52)	Hips (n = 52)	Knees (n = 52)	Ankles (n = 52)	Feet (n = 52)	Sacroiliac (n = 52)	Cervical (n = 26)	Lumbar (n = 26)	Dorsal (n = 26)
**Radiologist #1, first read**											
Joint effusion	6 (11.5)	15 (28.8)	20 (38.5)	1 (1.9)	24 (46.2)	32 (61.5)	20 (38.5)	0 (0.0)			
Bursitis	13 (25.0)	1 (1.9)	0 (0.0)	8 (15.4)	0 (0.0)	25 (48.1)	16 (30.8)	0 (0.0)			
Tenosynovitis	0 (0.0)	4 (7.7)	23 (44.2)	0 (0.0)	0 (0.0)	18 (34.6)	3 (5.8)	0 (0.0)			
BME-like signal	0 (0.0)	3 (5.8)	0 (0.0)	1 (1.9)	0 (0.0)	4 (7.7)	0 (0.0)	10 (19.2)	1 (3.8)	3 (11.5)	1 (3.8)
Muscle edema	0 (0.0)	0 (0.0)	0 (0.0)	0 (0.0)	0 (0.0)	8 (15.4)	0 (0.0)	0 (0.0)	0 (0.0)	0 (0.0)	0 (0.0)
Kager fat-pad edema						34 (65.4)					
**Radiologist #1, second read**											
Joint effusion	6 (11.5)	12 (23.1)	20 (38.5)	1 (1.9)	23 (44.2)	32 (61.5)	20 (38.5)	0 (0.0)			
Bursitis	10 (19.2)	1 (1.9)	0 (0.0)	7 (13.5)	0 (0.0)	25 (48.1)	14 (26.9)	0 (0.0)			
Tenosynovitis	0 (0.0)	4 (7.7)	19 (36.5)	0 (0.0)	0 (0.0)	16 (30.8)	3 (5.8)	0 (0.0)			
BME-like signal	0 (0.0)	3 (5.8)	0 (0.0)	1 (1.9)	0 (0.0)	4 (7.7)	0 (0.0)	10 (19.2)	1 (3.8)	3 (11.5)	1 (3.8)
Muscle edema	0 (0.0)	0 (0.0)	0 (0.0)	0 (0.0)	0 (0.0)	8 (15.4)	0 (0.0)	0 (0.0)	0 (0.0)	0 (0.0)	0 (0.0)
Kager fat-pad edema						31 (59.6)					
**Radiologist #2**											
Joint effusion	6 (11.5)	15 (28.8)	19 (36.5)	1 (1.9)	24 (46.2)	30 (57.7)	14 (26.9)	0 (0.0)			
Bursitis	13 (25.0)	1 (1.9)	0 (0.0)	8 (15.4)	0 (0.0)	25 (48.1)	12 (23.1)	0 (0.0)			
Tenosynovitis	0 (0.0)	4 (7.7)	20 (38.5)	0 (0.0)	0 (0.0)	15 (28.8)	3 (5.8)	0 (0.0)			
BME-like signal	0 (0.0)	3 (5.8)	0 (0.0)	1 (1.9)	0 (0.0)	4 (7.7)	0 (0.0)	10 (19.2)	1 (3.8)	4 (15.4)	1 (3.8)
Muscle edema	0 (0.0)	0 (0.0)	0 (0.0)	0 (0.0)	0 (0.0)	8 (15.4)	0 (0.0)	0 (0.0)	0 (0.0)	0 (0.0)	0 (0.0)
Kager fat-pad edema						32 (61.5)					

Data are expressed as n (%). **BME:** bone marrow edema.
